# Selenoprotein F Deficiency Drives Diet-Induced Metabolic Dysfunction in Female Mice by Aggravating Hypothalamic Endoplasmic Reticulum Stress

**DOI:** 10.3390/biology15131017

**Published:** 2026-06-26

**Authors:** Zimeng Li, Pengyu Zhao, Wanru Yang, Hongmei Liu

**Affiliations:** 1 Hubei Key Laboratory of Bioinorganic Chemistry and Materia Medica, School of Chemistry and Chemical Engineering, Huazhong University of Science and Technology, Wuhan 430074, China; d202280157@hust.edu.cn (Z.L.); d202580192@hust.edu.cn (W.Y.); 2 Key Laboratory of Molecular Biophysics of the Ministry of Education, College of Life Science and Technology, Huazhong University of Science and Technology, Wuhan 430074, China; pengyu122601@hust.edu.cn; 3 Hubei Engineering Research Center for Biomaterials and Medical Protective Materials, Wuhan 430074, China

**Keywords:** selenoprotein F, hypothalamus, endoplasmic reticulum stress, leptin resistance, thermogenesis

## Abstract

Obesity exhibits pronounced sex-dependent differences in susceptibility and progression; however, the molecular mechanisms coordinating central energy sensing with peripheral thermogenic responses remain incompletely defined. Selenoprotein F (SELENOF), an endoplasmic reticulum (ER)-resident member of the selenoprotein family involved in protein quality control and redox-sensitive metabolic regulation, has not previously been investigated in the context of diet-induced obesity. In the present study, WT and SELENOF-deficient mice subjected to a 16-week high-fat diet (HFD) were combined with primary brown adipocyte experiments to determine the role of SELENOF in systemic metabolic homeostasis. SELENOF deficiency markedly aggravated HFD-induced weight gain, adipose tissue expansion, dyslipidemia, and hyperleptinemia selectively in female mice, whereas no genotype-dependent effects were observed in males. Mechanistically, SELENOF deficiency intensified hypothalamic ER stress and leptin resistance, as reflected by increased GRP78, p-IRE1α, and p-PERK expression together with SOCS3 upregulation, reduced STAT3 phosphorylation, and activation of the IKK/NF-κB inflammatory pathway. In parallel, SELENOF deficiency reduced circulating free triiodothyronine (FT_3_) levels and the ratio of free triiodothyronine to free thyroxine (FT_3_/FT_4_ ratio), and suppressed DIO2 and UCP1 expression in brown adipose tissue (BAT). Experiments in primary brown adipocytes further showed that SELENOF deficiency did not disrupt proximal β3-adrenergic signaling but attenuated the downstream induction of DIO2 and UCP1. Collectively, these findings provide preliminary evidence that SELENOF is associated with sex-dependent metabolic adaptation during HFD-induced stress by linking hypothalamic proteostasis with the thyroid hormone-related thermogenic signaling program in BAT.

## Simple Summary

Obesity develops differently in females and males, but the biological reasons for this difference are still not fully understood. In this study, we examined whether selenoprotein F, a protein that helps cells maintain normal protein quality control, protects against metabolic problems caused by a high-fat diet. We found that the loss of selenoprotein F made female mice more vulnerable to weight gain, fat accumulation, abnormal blood lipid levels, and increased leptin, a hormone involved in the control of appetite and energy balance. These effects were much less evident in male mice. We also found signs of cellular stress in the hypothalamus, a brain region that helps regulate body weight, together with reduced activity of molecular pathways associated with heat production in brown fat. Our findings suggest that selenoprotein F may contribute to the coordination between brain-based energy regulation and brown-fat metabolism in a sex-dependent manner. This work provides a basis for future studies on why obesity develops differently in females and males.

## 1. Introduction

Obesity is now widely recognized as a complex, chronic systemic disease rather than merely the result of energy intake exceeding energy expenditure. Its pathogenesis involves the coordinated interplay of multiple processes, including dysregulation of central neural control, remodeling of peripheral metabolic tissues, chronic low-grade inflammation, endocrine imbalance, and disruption of organelle homeostasis [1,2,3,4]. Within this framework, the hypothalamus—situated at the nexus where nutritional status, adipokines, insulin signaling, and autonomic nervous system output converge—functions as a central regulator of energy homeostasis. Accordingly, structural and functional disturbances within hypothalamic circuits are increasingly recognized as key contributors to obesity pathogenesis [5,6].

Hypothalamic alterations in obesity do not represent a single inflammatory event but rather a progressive process involving inflammation, gliosis, extracellular matrix remodeling, and disruption of intracellular protein homeostasis. HFD exposure induces metabolic inflammation in key hypothalamic regions such as the arcuate nucleus, promotes glial activation, and remodels the local extracellular matrix, thereby impairing adaptive neuronal responses to changes in energy status [5,6,7]. Importantly, the endoplasmic reticulum (ER)-associated protein quality control system plays a pivotal role in maintaining leptin signaling integrity in POMC neurons, indicating that impaired hypothalamic proteostasis may represent an upstream mechanism contributing to diet-induced obesity [8]. Thus, extending the investigative focus from hypothalamic inflammation toward ER functional imbalance may provide deeper insight into the mechanisms initiating obesity.

Within the central metabolic regulatory network, leptin serves as a key signaling molecule linking peripheral adipose tissue status with intrinsic brain energy-regulatory programs. By acting on hypothalamic leptin receptors, leptin regulates food intake, energy expenditure, reward-related behavior, and neuroendocrine output, thereby maintaining systemic energy balance [9,10,11]. In obesity, however, neuronal responsiveness to leptin is markedly attenuated despite elevated circulating concentrations, resulting in leptin resistance that contributes to persistent metabolic dysregulation [12,13,14,15,16]. Because leptin resistance reflects coordinated alterations in post-receptor signaling, cellular stress responses, the glial microenvironment, and reward-system regulation [9,10,11,13], ER-resident regulators capable of influencing both proteostasis and leptin signaling sensitivity may function as mechanistic links between central stress responses and peripheral obesity phenotypes.

The selenoprotein family constitutes a functionally specialized group of proteins essential for maintaining redox balance, protein-folding quality control, and endocrine-metabolic regulation; several members are localized within the ER, where they participate in adaptive responses to metabolic stress [17,18,19,20]. Because selenium availability directly determines selenoprotein synthesis and activity, selenium-dependent pathways are increasingly recognized as modulators of metabolic adaptation under conditions of nutritional stress [21,22]. As an ER-resident selenoprotein, SELENOF participates in glycoprotein folding and quality control, suggesting a role in regulating ER homeostasis during metabolic challenge [17]. Deletion of SELENOF disrupts glucose metabolism and redox balance in vivo, indicating that SELENOF functions as a regulator of metabolic homeostasis rather than merely an antioxidant-associated component [23]. Furthermore, growing evidence links selenoproteins to adipocyte thermogenesis, thyroid function, and dysregulation of glucose and lipid metabolism [24,25]. Nevertheless, the role of SELENOF in diet-induced obesity, particularly its potential involvement in hypothalamic stress responses, leptin resistance, and peripheral thermogenic regulation, remains insufficiently characterized.

Obesity and its associated metabolic disorders exhibit pronounced sexual dimorphism. Differences between males and females in fat distribution, adipose tissue function, endocrine milieu, inflammatory responses, and susceptibility to metabolic stress contribute to distinct phenotypic trajectories [26,27,28,29,30]. Although traditionally attributed to sex hormones, increasing evidence indicates that adipose tissue plasticity, neuroendocrine coupling, and tissue-specific metabolic effector mechanisms also contribute to these differences [26,27,28,29,30]. Notably, sex-dependent variation in the thermogenic responsiveness of brown and beige adipose tissues may modulate the peripheral consequences of disturbances in central metabolic regulation [31,32]. Therefore, integrating sex as a biological variable is essential when evaluating the metabolic role of SELENOF.

Brown adipose tissue (BAT) serves as a major effector organ of adaptive thermogenesis and energy expenditure, integrating β-adrenergic stimulation, mitochondrial activity, and local thyroid hormone activation. Through DIO2-mediated conversion of thyroxine (T_4_) to triiodothyronine (T_3_), thyroid hormones promote the expression of thermogenic genes such as UCP1 and establish an efficient thermogenic program within BAT [33,34,35,36,37]. Impaired BAT function is closely associated with obesity and insulin resistance, highlighting thermogenic adipose tissue as a promising target for metabolic intervention [38,39,40,41]. Interactions between selenium, selenoproteins, and thermogenic adipocyte function further suggest that this protein family may coordinate thyroid hormone activation with the execution of thermogenic programs [24,25]. Based on these observations, we hypothesized that SELENOF ablation may simultaneously disrupt central energy sensing and thyroid hormone-dependent thermogenic activation in BAT.

Against this background, the present study proposes that SELENOF contributes to systemic energy homeostasis during HFD exposure by maintaining hypothalamic proteostasis and leptin signaling sensitivity while preserving local thyroid hormone activation and the thermogenic signaling program in BAT. Given the established sex differences in both obesity and BAT biology, we further investigated whether SELENOF deficiency exacerbates hypothalamic ER stress, leptin resistance, and inflammatory signaling and disrupts the DIO2-UCP1 axis in a sex-dependent manner, thereby promoting metabolic disturbances particularly in females. Elucidating these mechanisms may provide new insight into how ER-resident selenoproteins participate in obesity regulation and help define questions for future sex-informed metabolic studies.

## 2. Materials and Methods

### 2.1. Animals

Wild-type (WT) C57BL/6J mice were obtained from Henan Skobes Biotechnology Co., Ltd. (Anyang, China). SELENOF knockout (KO) C57BL/6J mice generated using the CRISPR/Cas9 system were established and maintained in our facility. Genotypic and phenotypic validation of the KO model was performed by agarose gel electrophoresis and DNA sequencing, as described in the Supplementary Methods [42]. Detailed validation data are provided in Figure S1. Animals were housed in groups of five per cage under controlled environmental conditions (22 ± 2 °C; 12 h light/dark cycle) with ad libitum access to standard chow and water. Health status and behavior were monitored daily throughout the study. All experimental procedures were approved by the Institutional Animal Care and Use Committee of Huazhong University of Science and Technology (HUST-IACUC-2026-0021).

At six weeks of age, male and female mice were analyzed as separate experimental cohorts. For each sex, WT and KO mice were randomly allocated to four experimental groups before dietary intervention: WT + ND, WT + HFD, KO + ND, and KO + HFD (*n* = 8–10 mice per sex-specific group). Mice were fed either a normal diet (ND; 10% kcal from fat) or a high-fat diet (HFD; 60% kcal from fat; D12492, Research Diets) for 16 weeks, until 22 weeks of age. Body weight was recorded weekly, and metabolic, biochemical, and molecular analyses were performed at predetermined time points. Estrous-cycle synchronization and systematic estrous-cycle monitoring were not performed in female mice.

### 2.2. Glucose Tolerance Tests and Insulin Tolerance Tests

For glucose tolerance tests (GTT), mice were fasted for 12 h and then given glucose (1 g/kg body weight) by oral gavage. For insulin tolerance tests (ITT), mice were fasted for 4 h and then injected intraperitoneally with insulin (Yuanye Biotechnology, Shanghai, China) at sex-specific doses (0.8 U/kg for males; 0.6 U/kg for females). Blood glucose levels were measured at indicated time points using a portable glucometer (ACCU-CHEK Advantage II, Roche Diagnostics, Indianapolis, IN, USA). All tests were performed at 20–21 weeks of age.

### 2.3. Sample Preparation

After 16 weeks of dietary intervention, mice were fasted for 12 h and then euthanized. Under anesthesia, blood samples were collected by eyeball enucleation. Serum was separated by centrifugation (1000× *g*, 20 min, 4 °C) and stored at −80 °C until analysis. Brown adipose tissue (BAT) and multiple white adipose tissue (WAT) depots were carefully excised. The WAT depots included gonadal adipose tissue (referred to as ovarian WAT [ovWAT] in females and epididymal WAT [eWAT] in males), inguinal WAT (iWAT), mesenteric WAT, and perirenal WAT. Gonadal and inguinal WAT depots were weighed immediately after dissection, and their weights were normalized to total body weight and expressed as percentages. For subsequent analyses, tissue samples were either snap-frozen in liquid nitrogen and stored at −80 °C for molecular assays or fixed in 4% paraformaldehyde for histopathological evaluation.

### 2.4. Serum Biochemical Analysis

Serum concentrations of triglycerides (TG), total cholesterol (TC), free fatty acids (FFA), and leptin were measured using commercial assay kits from Elabscience (Wuhan, China; catalog numbers: E-BC-K261-M for TG, E-BC-K109-M for TC, E-BC-K013-M for FFA, and E-EL-M3008 for leptin) according to the manufacturers’ instructions. Serum alanine aminotransferase (ALT) and aspartate aminotransferase (AST) activities were determined using assay kits from the Nanjing Jiancheng Bioengineering Institute (Nanjing, China; product codes: C009-2-1 for ALT and C010-2-1 for AST). Serum estradiol (E2; Elabscience, E-OSEL-M0008), thyroid-stimulating hormone (TSH; Solarbio, SEKM-0272, Beijing, China), free thyroxine (FT_4_; Elabscience, E-EL-0122), and free triiodothyronine (FT_3_; Elabscience, E-EL-0079) concentrations were determined via enzyme-linked immunosorbent assay (ELISA).

### 2.5. Histopathological Analysis of Adipose Tissue

Adipose tissues fixed in 4% paraformaldehyde (Servicebio, Wuhan, China) were dehydrated through graded ethanol, cleared in xylene, and embedded in paraffin. Sections (4–5 μm) were deparaffinized, rehydrated, and stained with hematoxylin and eosin (Servicebio). Stained sections were examined by light microscopy. Representative images were selected to illustrate adipose tissue morphology. Adipocyte size was determined using ImageJ 1.52v software, measuring a minimum of 100 cells per group.

### 2.6. Western Blot Analysis

Protein lysates from WAT, BAT, hypothalamus, and isolated brown adipocytes were prepared using RIPA buffer supplemented with protease inhibitors (Solarbio, Beijing, China). Total protein concentrations were determined using a BCA protein assay kit (Elabscience). Equal amounts of protein were separated by SDS-PAGE and transferred onto PVDF membranes (Millipore, Burlington, MA, USA). Membranes were blocked with blocking buffer (Boster, Wuhan, China) and incubated overnight at 4 °C with primary antibodies against the proteins of interest. Membranes were then incubated for 1 h at room temperature with HRP-conjugated secondary antibodies (Biosharp, Beijing, China). Protein signals were detected using an enhanced chemiluminescence substrate kit (Millipore) and visualized with a Tanon 5200 MultiImage System (Tanon, Shanghai, China). Band intensities were quantified using ImageJ software. Target protein expression levels were normalized to β-actin or HSP90 as internal controls. For Western blot analyses of animal tissues, three independent biological tissue samples were analyzed per group, and each lane represented protein lysate obtained from an individual mouse.

Primary antibodies included ACC (3662, 1:1000), FAS (3180, 1:1000), HSL (4107, 1:1000), IRE1α (3294, 1:1000), phospho-p65 (Ser536, 3033, 1:1000), and p65 (8242, 1:1000) (Cell Signaling Technology, Danvers, MA, USA); ATGL (A5126, 1:1000), phospho-PERK (Thr982, AP1501, 1:1000), PERK (A27664, 1:1000), UCP1 (A21979, 1:1000), and β-actin (AC026, 1:50,000) (ABclonal, Wuhan, China); GRP78 (ab108615, 1:1000) (Abcam, Cambridge, UK); ATF6 (WL01153, 1:1000), ObRb (WL0162a, 1:1000), SOCS3 (WL01364, 1:1000), phospho-STAT3 (Ser727, WL06214, 1:1000), STAT3 (WL01836, 1:1000), phospho-IKKα/β (Ser176/180, WLA0347, 1:1000), IKKα/β (WL01900, 1:1000), phospho-CREB1 (Ser133, WLA0644, 1:1000; Wanleibio), CREB1 (WL01848, 1:1000), HSP90 (WL01763, 1:1000) (Wanleibio, Shenyang, China); leptin (DF8583, 1:500) (Affinibody, Wuhan, China); phospho-IRE1α (Ser724, AF5842, 1:1000) (Beyotime, Shanghai, China); DIO2 (CSB-PA440740, 1:1000) (Cusabio, Wuhan, China), and SELENOF (1:500) (BOSTER, Wuhan, China).

### 2.7. RNA Extraction and Quantitative Real-Time PCR for Leptin mRNA in Adipose Tissue

Total RNA was extracted from 100 mg of ovWAT using TRIzol™ reagent (Thermo Fisher Scientific, Waltham, MA, USA). RNA concentration was determined spectrophotometrically. For reverse transcription, 5 μg of total RNA was converted to cDNA using the RevertAid First Strand cDNA Synthesis Kit (Thermo Fisher Scientific) with oligo (dT) primers.

Quantitative real-time PCR was performed on a StepOne™ system (Applied Biosystems, Foster City, CA, USA) using SYBR^®^ Green Realtime PCR Master Mix (TOYOBO, Osaka, Japan). Primer sequences are listed in Table S1. β-Actin served as the internal reference gene. Relative gene expression levels were calculated using the 2^−ΔΔCt^ method.

### 2.8. Isolation, Differentiation, and Treatment of Primary Brown Adipocytes

Primary brown adipocytes were isolated from interscapular BAT of WT and SELENOF KO C57BL/6 pups aged 5–10 days (*n* = 5 per genotype). After cervical dislocation, pups were disinfected with 75% ethanol. Interscapular BAT depots were aseptically excised and immediately transferred into a 5 mL penicillin vial containing D-Hank’s balanced salt solution. The tissue was rinsed two to three times with D-Hank’s solution to remove residual blood and connective tissue and then minced into fragments of approximately 1 mm^3^ using sterile ophthalmic scissors. The minced tissue was transferred to a 6 cm culture dish containing 2 mL digestion buffer consisting of Hanks’ balanced salt solution supplemented with 0.2% type IV collagenase (Yuanye Biotechnology) and 10% bovine serum albumin (BSA; Macklin, Shanghai, China) and incubated for approximately 15 min at 37 °C with gentle shaking until a suspension of single cells and small clusters was observed microscopically. Digestion was terminated by adding an equal volume of DMEM/F12 medium (Pricella Biotechnology, Wuhan, China) containing 10% fetal bovine serum (FBS, Tianhang Biotechnology Co., Ltd., Huzhou, China). The suspension was filtered through a 40 μm cell strainer (Biosharp) and centrifuged at 1200 rpm for 5 min. The stromal vascular fraction (SVF) pellet was resuspended in DMEM/F12 medium supplemented with 10% FBS, gently pipetted to disperse cells, counted, and seeded at a density of 2–3 × 10^5^ cells/cm^2^. Cells were cultured at 37 °C in a humidified incubator with 5% CO_2_.

After reaching confluence, cells were maintained for an additional 48 h to achieve growth synchronization before the induction of adipogenic differentiation. Differentiation was initiated by incubation for 48 h in Induction Medium I consisting of DMEM/F12, 10% FBS, 1 nM T_3_ (Macklin, Shanghai, China), 1 μg/mL insulin (Yuanye Biotechnology), 0.125 mM indomethacin (Merck KGaA, Darmstadt, Germany), and 0.5 mM isobutylmethylxanthine (IBMX; Macklin). Cells were then cultured for 96 h in Induction Medium II consisting of DMEM/F12, 10% FBS, 1 nM T_3_, and 1 μg/mL insulin. Successful differentiation was confirmed by lipid droplet accumulation in more than 80% of cells.

To evaluate thermogenic responsiveness, mature brown adipocytes were treated with 1 μM CL316,243 (GlpBio, Montclair, CA, USA), a selective β3-adrenergic receptor agonist, or vehicle control (DMSO, Biosharp) for either 30 min (analysis of phosphorylated signaling proteins) or 6 h (analysis of downstream gene expression), after which cells were collected for further analysis.

### 2.9. Statistical Analysis

Statistical analyses were performed separately for the male and female datasets. Data processing and visualization were conducted using GraphPad Prism (version 8). Differences among multiple groups were evaluated using one-way analysis of variance (ANOVA) followed by Tukey’s post hoc test. Results are presented as mean ± standard deviation (SD), and statistical significance was defined as *p* < 0.05.

## 3. Results

### 3.1. SELENOF Deficiency Exacerbates HFD-Induced Obesity in Female Mice

**Figure 1:**
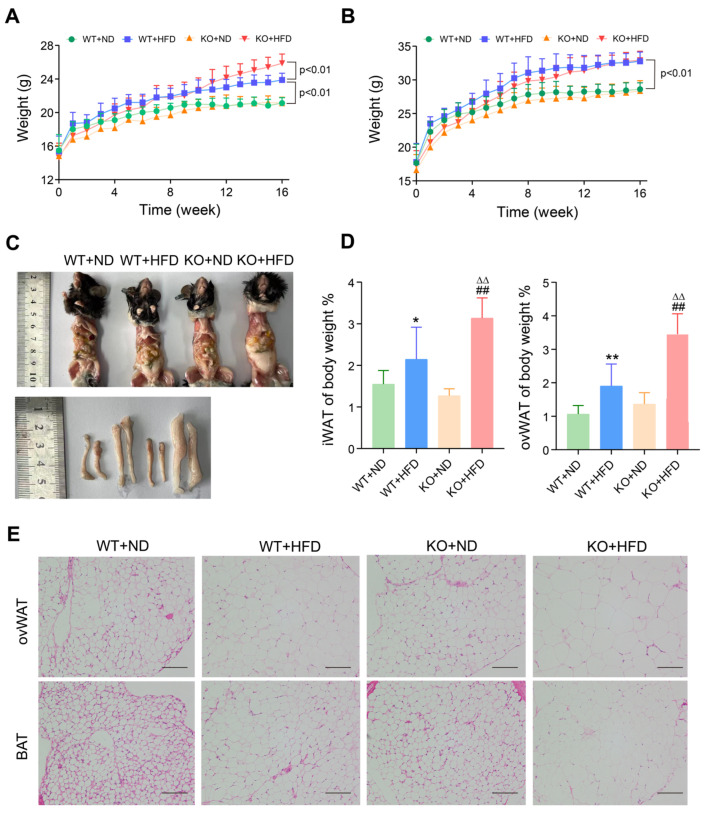
SELENOF deficiency exacerbates HFD-induced body weight gain, white adipose expansion, and adipocyte hypertrophy in female mice, with no effect in males. (**A**,**B**) Body weight curves of WT and SELENOF KO mice fed a normal diet (ND) or high-fat diet (HFD) for 16 weeks: (**A**) Female mice and (**B**) male mice. (**C**) Representative images of ovarian white adipose tissue (ovWAT) and inguinal white adipose tissue (iWAT) depots from female mice. (**D**) ovWAT-to-body-weight and iWAT-to-body-weight ratios. (**E**) Representative hematoxylin and eosin (H&E) staining sections of ovWAT and brown adipose tissue (BAT) from female mice. SELENOF deficiency enhanced adipocyte hypertrophy in ovWAT and promoted BAT whitening (increased lipid droplet accumulation) under HFD conditions. Scale bar, 100 μm. Data are presented as mean ± SD (*n* = 10 per group). Statistical differences were evaluated using one-way ANOVA followed by Tukey’s post hoc test. * *p* < 0.05 and ** *p* < 0.01 vs. WT + ND; ^##^ *p* < 0.01 vs. KO + ND; ^ΔΔ^ *p* < 0.01 vs. WT + HFD.

To determine whether SELENOF deficiency influences susceptibility to diet-induced obesity, body weight progression was monitored in WT and KO mice during a 16-week dietary intervention. Under ND conditions, body weight did not differ between WT and KO animals of either sex. HFD feeding induced progressive weight gain in both genotypes; however, female KO mice exhibited significantly greater body weight gain than WT animals throughout the intervention period. In contrast, no genotype-dependent differences were observed in male mice under HFD conditions, indicating a sex-specific metabolic response to SELENOF deficiency (Figure 1A,B).

To further evaluate adipose tissue expansion, ovWAT and iWAT depots were analyzed in female mice after 16 weeks of dietary intervention. Representative depot images demonstrated pronounced enlargement of adipose tissue following HFD feeding, with visibly greater expansion in KO animals compared with WT controls. Organ coefficient analysis showed that HFD significantly increased the relative weight (tissue/body weight) of both ovWAT and iWAT in WT and KO mice. Furthermore, the organ coefficients of both depots remained significantly higher in KO + HFD animals compared with WT + HFD controls (Figure 1C,D).

In contrast, analysis of adipose depots in male mice demonstrated that, although HFD feeding significantly increased eWAT and iWAT mass relative to ND controls, no genotype-dependent differences were observed between WT and KO animals (Figure S2A,B).

Histological examination further revealed marked sex-dependent differences in adipose tissue morphology. In female mice, ovWAT from ND groups displayed regular adipocyte morphology with uniform cellular organization, whereas HFD feeding induced pronounced adipocyte hypertrophy that was further accentuated in KO animals. A similar pattern was observed in BAT, where lipid droplet enlargement was substantially greater in KO mice than in WT controls under HFD conditions, indicating enhanced BAT whitening in the absence of SELENOF (Figure 1E). Quantitative analysis of cell size confirmed that HFD significantly increased adipocyte area in both ovWAT and BAT of female mice, and SELENOF deficiency further exacerbated this enlargement (Figure S3A,B). In male mice, adipocyte enlargement occurred in response to HFD feeding independently of genotype, with no additional morphologic differences between WT and KO animals were detected in either eWAT or BAT (Figure S2C). These morphologic observation was further supported by cell size quantification (Figure S3C,D).

### 3.2. SELENOF Deficiency Does Not Alter Glucose Tolerance or Insulin Sensitivity

**Figure 2:**
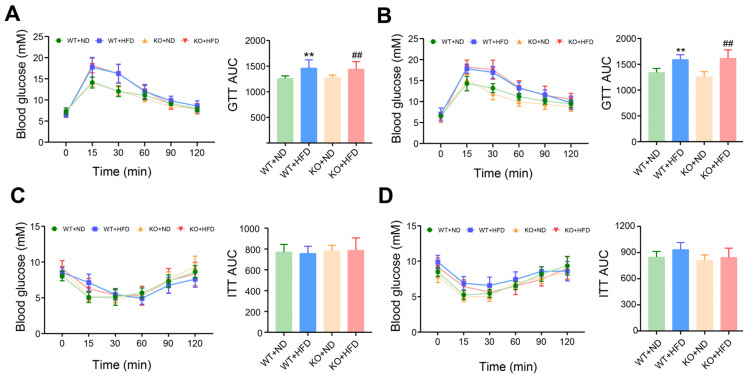
Effects of SELENOF deficiency on glucose tolerance and insulin sensitivity after 16 weeks of dietary intervention. (**A**) Glucose tolerance test (GTT) curves and area-under-the-curve (AUC) values in female mice. (**B**) GTT curves and AUC values in male mice. (**C**) Insulin tolerance test (ITT) curves and AUC values in female mice. (**D**) ITT curves and AUC values in male mice. HFD feeding impaired glucose tolerance independently of genotype, whereas insulin sensitivity remained comparable between WT and SELENOF KO mice of both sexes. Data are presented as mean ± SD (*n* = 8–10 per group). AUC values were analyzed using one-way ANOVA followed by Tukey’s post hoc test. ** *p* < 0.01 vs. WT + ND; ^##^ *p* < 0.01 vs. KO + ND.

To determine whether SELENOF deficiency influences systemic glucose homeostasis under HFD conditions, GTT and ITT were performed after 14 weeks of dietary intervention. HFD feeding significantly impaired glucose tolerance in both WT and SELENOF KO mice compared with animals maintained on a normal diet, indicating that prolonged exposure to HFD disrupts physiological glucose handling capacity. However, no genotype-dependent differences were observed between WT and KO mice under either dietary condition in female or male cohorts (Figure 2A,B). Similarly, assessment of insulin sensitivity revealed no statistically significant differences between WT and KO mice in either sex. Although HFD feeding produced mild alterations in insulin responsiveness compared with normal diet controls, the absence of SELENOF did not further modify insulin tolerance responses, indicating that SELENOF deficiency does not contribute to systemic insulin resistance under the experimental conditions used in this study (Figure 2C,D).

Together, these findings demonstrate that, despite the pronounced adiposity phenotype observed in female KO mice, SELENOF deficiency does not impair whole-body glucose tolerance or insulin sensitivity, suggesting that the metabolic effects of SELENOF loss are primarily restricted to adipose tissue remodeling rather than systemic glucose regulation.

### 3.3. SELENOF Deficiency Aggravates Dyslipidemia and Endocrine Imbalance in Female Mice

**Figure 3:**
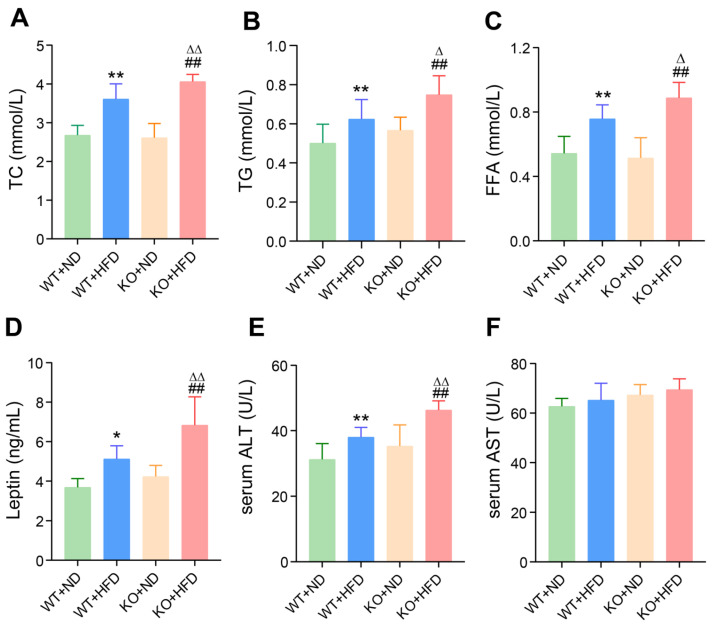
Effects of SELENOF deficiency on serum biochemical parameters in female mice following 16 weeks of HFD intervention. (**A**) Total cholesterol (TC). (**B**) Triglycerides (TG). (**C**) Free fatty acids (FFA). (**D**) Leptin. (**E**) Alanine aminotransferase (ALT). (**F**) Aspartate aminotransferase (AST). SELENOF deficiency significantly aggravated HFD-induced dyslipidemia, adipose endocrine activation, and hepatocellular stress in female mice. Data are presented as mean ± SD (*n* = 8–10 per group). Statistical differences were evaluated using one-way ANOVA followed by Tukey’s post hoc test. * *p* < 0.05 and ** *p* < 0.01 vs. WT + ND; ^##^ *p* < 0.01 vs. KO + ND; ^Δ^ *p* < 0.05 and ^ΔΔ^ *p* < 0.01 vs. WT + HFD.

To further determine whether SELENOF deficiency contributes to systemic metabolic dysregulation under HFD, serum lipid parameters, adipose-derived endocrine markers, and indicators of hepatic function were evaluated after 16 weeks of dietary intervention. HFD feeding significantly increased circulating levels of TC, TG, FFA, and leptin in female mice compared with animals maintained on a normal diet, confirming that prolonged HFD exposure induced pronounced disturbance of systemic lipid homeostasis accompanied by activation of adipose endocrine signaling. Importantly, SELENOF deficiency further aggravated these metabolic alterations. Relative to WT + HFD mice, KO + HFD mice exhibited significantly higher serum TC, TG, FFA, and leptin levels, indicating enhanced lipid mobilization, increased adipose tissue dysfunction and amplification of obesity-associated endocrine imbalance (Figure 3A–D). In addition to dyslipidemia, SELENOF deficiency intensified markers of hepatic stress. Serum ALT activity was significantly elevated in KO + HFD mice compared with WT + HFD controls, whereas AST levels remained unchanged between genotypes, suggesting selective exacerbation of HFD-induced hepatocellular injury rather than generalized liver dysfunction (Figure 3E,F).

In contrast to the pronounced metabolic phenotype observed in female mice, SELENOF deficiency did not further modify serum lipid parameters or liver enzyme activity in male mice. Although HFD feeding significantly increased circulating TC, TG, FFA, leptin, ALT, and AST levels relative to normal diet controls, no statistically significant differences between WT + HFD and KO + HFD groups were detected for any of the analyzed parameters (Figure S4).

These findings indicate that the regulatory role of SELENOF in systemic lipid metabolism exhibits marked sexual dimorphism, characterized by a protective function against diet-induced dyslipidemia and adipose endocrine dysfunction specifically in female mice but not in males.

### 3.4. SELENOF Deficiency Impairs Lipid Mobilization and Increases Leptin Production in WAT of Female Mice

**Figure 4:**
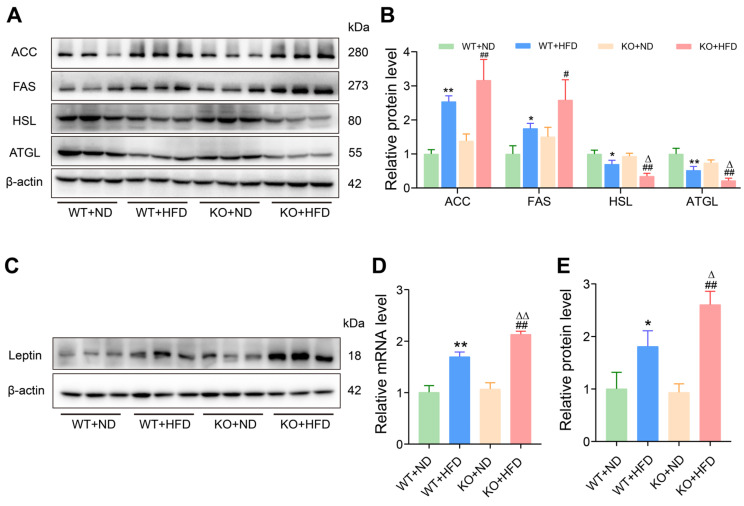
SELENOF deficiency suppresses lipolysis and enhances leptin expression in ovarian white adipose tissue (ovWAT) of female mice following 16 weeks of HFD intervention. (**A**) Representative Western blot analysis of ACC, FAS, HSL, and ATGL in ovWAT. (**B**) Quantification of protein expression normalized to β-actin. SELENOF deficiency further reduced expression of lipolytic enzymes under HFD conditions without additional effects on lipogenic regulators. (**C**) Western blot analysis of leptin protein levels in ovWAT. (**D**) Quantification of leptin protein expression normalized to β-actin. (**E**) Relative leptin mRNA expression measured by quantitative real-time PCR. Data are presented as mean ± SD (*n* = 3 independent biological tissue samples from individual mice per group). Statistical differences were evaluated using one-way ANOVA followed by Tukey’s post hoc test. * *p* < 0.05 and ** *p* < 0.01 vs. WT + ND; ^#^ *p* < 0.05 and ^##^ *p* < 0.01 vs. KO + ND; ^Δ^ *p* < 0.05 and ^ΔΔ^ *p* < 0.01 vs. WT + HFD. The original Western blot images are summarized in File S1.

To determine whether enhanced adiposity observed in female SELENOF-deficient mice was associated with altered lipid metabolic regulation within WAT, the expression of key enzymes controlling lipogenesis and lipolysis was examined in ovWAT following 16 weeks of dietary intervention. Consistent with the development of diet-induced adipose expansion, HFD feeding significantly increased the expression of the lipogenic enzymes acetyl-CoA carboxylase (ACC) and fatty acid synthase (FAS) in WT animals, confirming activation of lipid synthesis pathways under HFD conditions. However, SELENOF deficiency did not further enhance the expression of these lipogenic regulators, indicating that the increased adiposity observed in KO mice was not driven by additional stimulation of de novo lipogenesis (Figure 4A,B).

In contrast, analysis of lipolytic pathways revealed a pronounced suppression of lipid mobilization in SELENOF-deficient animals. Expression of hormone-sensitive lipase (HSL) and adipose triglyceride lipase (ATGL) was significantly reduced following HFD feeding in WT mice and declined even further in KO animals, demonstrating that SELENOF deficiency selectively aggravates the diet-induced impairment of lipolysis (Figure 4A,B). These findings indicate that reduced triglyceride hydrolysis rather than enhanced lipid synthesis represents the primary mechanism contributing to excessive adipose tissue accumulation in SELENOF-deficient female mice.

Because adipose tissue expansion is closely associated with endocrine remodeling of metabolic signaling networks, leptin expression was next evaluated in ovWAT of female mice. HFD feeding significantly increased both leptin protein and mRNA levels, indicating activation of adipose-derived endocrine signaling in response to chronic nutrient excess. Notably, SELENOF deficiency further amplified leptin expression at both mRNA and protein levels, suggesting that loss of SELENOF promotes adipose endocrine dysregulation (Figure 4C–E).

### 3.5. SELENOF Deficiency Enhances Hypothalamic ER Stress and Promotes Leptin Resistance in Female Mice

**Figure 5:**
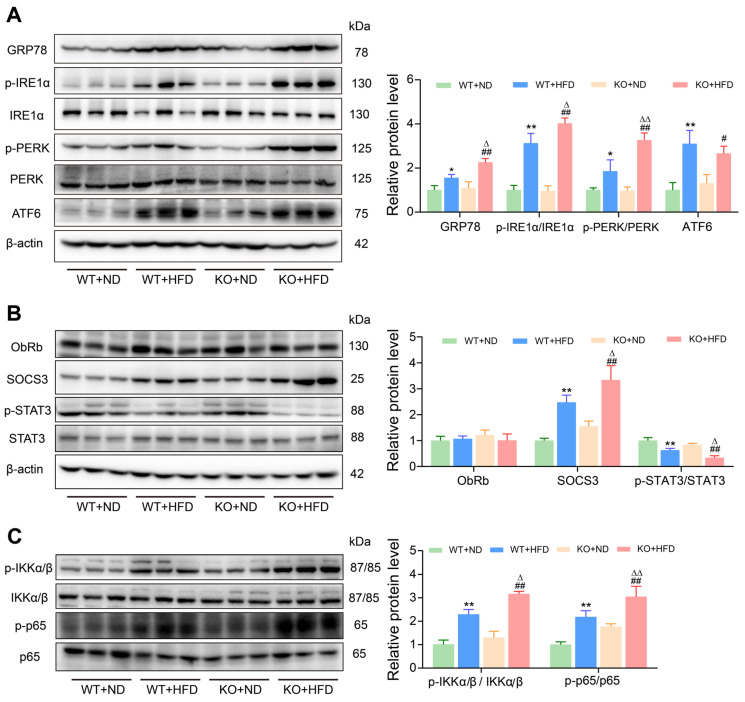
SELENOF deficiency exacerbates hypothalamic endoplasmic reticulum (ER) stress, leptin resistance, and inflammatory signaling in female mice following 16 weeks of HFD intervention. (**A**) Western blot analysis of GRP78, p-IRE1α, total IRE1α, p-PERK, total PERK, and ATF6 in hypothalamic tissue. p-IRE1α and p-PERK were normalized to their respective total proteins; GRP78 and ATF6 were normalized to β-actin. SELENOF deficiency further enhanced activation of ER stress pathways under HFD conditions. (**B**) Western blot analysis of ObRb, SOCS3, p-STAT3, and total STAT3. p-STAT3 was normalized to total STAT3; ObRb and SOCS3 were normalized to β-actin. SELENOF deficiency further increased SOCS3 expression and reduced STAT3 phosphorylation under HFD conditions, suggesting exacerbated hypothalamic leptin resistance. (**C**) Western blot analysis of p-IKKα/β, total IKKα/β, p-p65, and total p65. Phosphorylation levels were normalized to their respective total proteins. SELENOF deficiency further enhanced activation of the IKK/NF-κB inflammatory pathway under HFD conditions. Data are presented as mean ± SD (*n* = 3 independent biological tissue samples from individual mice per group). Statistical differences were evaluated using one-way ANOVA followed by Tukey’s post hoc test. * *p* < 0.05 and ** *p* < 0.01 vs. WT + ND; ^#^ *p* < 0.05 and ^##^ *p* < 0.01 vs. KO + ND; ^Δ^ *p* < 0.05 and ^ΔΔ^ *p* < 0.01 vs. WT + HFD. The original Western blot images are summarized in File S1.

Because SELENOF is an ER-resident protein, its loss may contribute to disturbances in central metabolic regulation within the hypothalamus by enhancing ER stress signaling under HFD conditions. To examine this possibility, the expression of key ER stress-related proteins was analyzed in hypothalamic tissue of female mice after 16 weeks of dietary intervention. The results demonstrated that HFD significantly increased the protein levels of the canonical ER stress markers GRP78, p-IRE1α, p-PERK, and ATF6 in hypothalamic tissue compared with normal diet controls, indicating activation of the unfolded protein response. Importantly, compared with the WT + HFD group, the protein levels of these ER stress markers were further elevated in the KO + HFD group, with GRP78, p-IRE1α, and p-PERK showing particularly pronounced increases (Figure 5A). These findings suggest that SELENOF deficiency aggravates diet-induced hypothalamic ER stress and supports a protective role of SELENOF in maintaining ER proteostasis under HFD conditions.

The hypothalamus serves as a central regulatory hub for systemic energy homeostasis, and persistent activation of ER stress signaling within this region is known to interfere with leptin receptor signaling and promote leptin resistance [43,44]. To investigate whether SELENOF deficiency affects hypothalamic leptin signaling pathways, the expression of the long leptin receptor isoform (ObRb) and its downstream signaling mediators was examined. There were no significant differences in hypothalamic ObRb protein levels among the experimental groups. However, HFD feeding significantly increased the expression of suppressor of cytokine signaling-3 (SOCS3), a negative regulator of leptin signaling, while simultaneously reducing phosphorylation of STAT3. Notably, SELENOF KO further enhanced SOCS3 expression and produced a more pronounced reduction in p-STAT3 levels compared with WT + HFD animals. These findings indicate that SELENOF deficiency aggravates diet-induced impairment of hypothalamic leptin signaling primarily through SOCS3-mediated inhibition of STAT3 activation rather than through changes in receptor abundance (Figure 5B).

ER stress within the hypothalamus can further promote inflammatory signaling through activation of the IKK/NF-κB pathway, which represents an important mechanism contributing to diet-induced central leptin resistance [45]. To examine whether SELENOF deficiency influences this pathway, the protein levels of phosphorylated IKKα/β and phosphorylated NF-κB p65 were analyzed. The results demonstrated that HFD feeding significantly activated hypothalamic IKK/NF-κB signaling, as reflected by increased phosphorylation of both IKKα/β and p65. Importantly, SELENOF deficiency further enhanced this activation compared with WT + HFD animals (Figure 5C).

### 3.6. SELENOF Deficiency Suppresses Thyroid Hormone-Related Thermogenic Signaling Independently of Circulating Estradiol Levels in Female Mice

To investigate whether the sex-specific metabolic phenotype observed in SELENOF-deficient female mice could be explained by alterations in circulating estrogen levels, serum estradiol concentrations were first examined. The results indicated that there were no significant differences in serum estradiol levels between WT and SELENOF KO female mice under HFD conditions (Figure S5).

**Figure 6:**
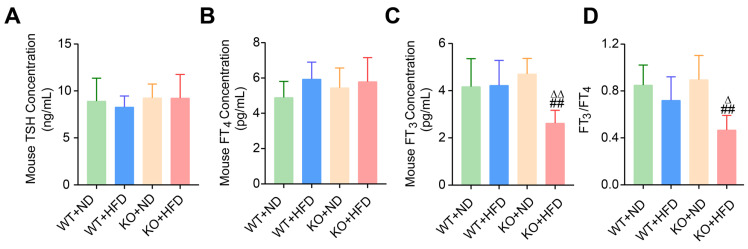
Effects of SELENOF deficiency on systemic thyroid hormone homeostasis in female mice following 16 weeks of HFD intervention. (**A**) Serum thyroid-stimulating hormone (TSH) levels. (**B**) Serum free thyroxine (FT_4_) levels. (**C**) Serum free triiodothyronine (FT_3_) levels. (**D**) FT_3_/FT_4_ ratio, defined as the ratio of free triiodothyronine to free thyroxine. SELENOF deficiency selectively reduced FT_3_ concentrations and the FT_3_/FT_4_ ratio under HFD conditions and also impaired peripheral thyroid hormone activation. Data are presented as mean ± SD (*n* = 8–10 per group). Statistical differences were evaluated using one-way ANOVA followed by Tukey’s post hoc test. ^##^ *p* < 0.01 vs. KO + ND; ^Δ^ *p* < 0.05 and ^ΔΔ^ *p* < 0.01 vs. WT + HFD.

Given the absence of detectable changes in circulating estradiol levels, additional endocrine mechanisms potentially contributing to the sex-specific metabolic phenotype were examined, with particular attention to thyroid hormone homeostasis. Because thyroid hormone activation depends on deiodinases belonging to the selenoprotein family, SELENOF deficiency was hypothesized to influence systemic thyroid hormone metabolism. Serum concentrations of TSH did not differ significantly among experimental groups, indicating preserved hypothalamic–pituitary–thyroid axis regulation (Figure 6A). Similarly, circulating FT_4_ levels remained unchanged (Figure 6B). In contrast, significant intergroup differences were observed in FT_3_ concentrations. Notably, HFD markedly reduced FT_3_ levels in SELENOF-deficient mice compared with both KO + ND and WT + HFD animals (Figure 6C). Analysis of the FT_3_/FT_4_ ratio further demonstrated that this reduction primarily reflected impaired peripheral conversion of FT_4_ to FT_3_ rather than decreased thyroid hormone synthesis (Figure 6D).

**Figure 7:**
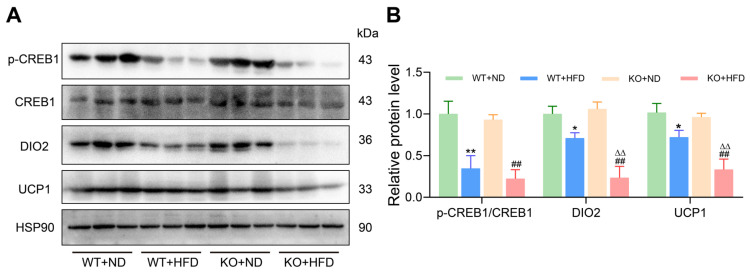
Effects of SELENOF deficiency on thermogenic signaling in brown adipose tissue (BAT) of female mice following HFD feeding. (**A**) Western blot analysis of p-CREB1, total CREB1, DIO2, and UCP1 in BAT. (**B**) Quantification of protein expression. p-CREB1 levels were normalized to total CREB1; DIO2 and UCP1 were normalized to HSP90. SELENOF deficiency further reduced DIO2 and UCP1 expression induced by HFD, without affecting p-CREB1 levels. Data are presented as mean ± SD (*n* = 3 independent biological tissue samples from individual mice per group). Statistical differences were evaluated using one-way ANOVA followed by Tukey’s post hoc test. * *p* < 0.05 and ** *p* < 0.01 vs. WT + ND; ^##^ *p* < 0.01 vs. KO + ND; ^ΔΔ^ *p* < 0.01 vs. WT + HFD. The original Western blot images are summarized in File S1.

Because BAT is a major site of local T_3_ production and thermogenic regulation, we examined DIO2, the key enzyme that converts T_4_ to active T_3_, in this tissue. Western blot analysis revealed that HFD reduced DIO2 protein expression in BAT of WT mice. Under the same dietary conditions, SELENOF deficiency further decreased DIO2 expression relative to WT + HFD animals, suggesting that SELENOF loss enhances diet-induced sup-pression of local thyroid hormone activation in thermogenic tissue. In contrast, phosphorylation of CREB1, a key downstream component of β-adrenergic signaling, was reduced in BAT by HFD but did not differ between WT and KO mice, suggesting that upstream adrenergic signaling remained largely preserved (Figure 7). At the molecular level, expression of the thermogenic marker UCP1 was reduced in BAT of WT mice under HFD conditions and further decreased in SELENOF KO animals, supporting aggravated sup-pression of the BAT thermogenic signaling program.

### 3.7. SELENOF Deficiency Attenuates Induction of the Thermogenic Program in β3-Adrenergically Stimulated Brown Adipocytes

**Figure 8:**
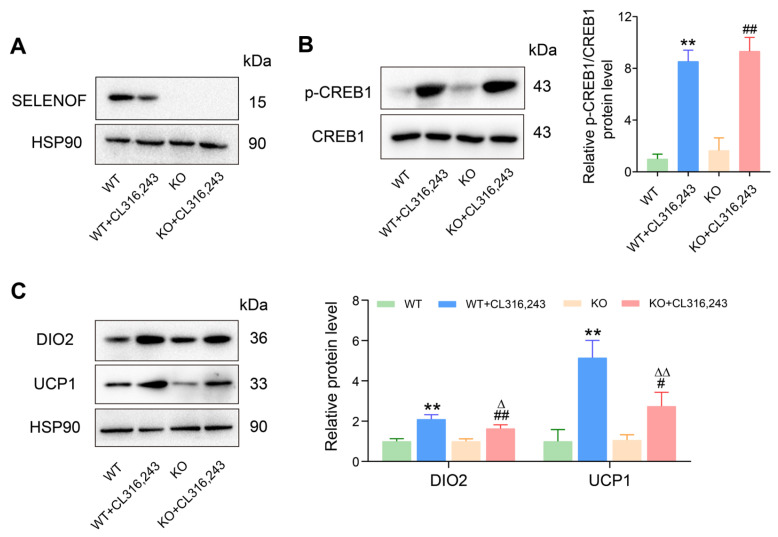
SELENOF deficiency attenuates β3-adrenergic-induced DIO2 and UCP1 expression without affecting early CREB1 signaling in primary brown adipocytes. (**A**) Western blot analysis of SELENOF protein levels in primary brown adipocytes after 6 h stimulation with CL316,243 (1 μM). (**B**) Western blot analysis of p-CREB1 and total CREB1 after 30 min of stimulation with CL316,243 (1 μM). Quantification of p-CREB1 was normalized to total CREB1. (**C**) Western blot analysis of DIO2 and UCP1 after 6 h of stimulation with CL316,243 (1 μM). Quantification of DIO2 and UCP1 was normalized to HSP90. SELENOF deficiency inhibited CL316,243-induced increase in DIO2 and UCP1 expression in primary brown adipocytes, without affecting p-CREB1 levels. Data are presented as mean ± SD from three experimental replicates per condition (*n* = 3). Statistical differences were evaluated using one-way ANOVA followed by Tukey’s post hoc test. ** *p* < 0.01 vs. WT; ^#^ *p* < 0.05 and ^##^ *p* < 0.01 vs. KO + CL316,243; ^Δ^ *p* < 0.05 and ^ΔΔ^ *p* < 0.01 vs. WT + CL316,243. The original Western blot images are summarized in File S1.

To confirm the efficacy of SELENOF gene KO in primary brown adipocytes and to determine whether its expression is regulated by β-adrenergic receptor signaling, SELENOF protein levels were examined under basal conditions and following stimulation with the selective β3-adrenergic receptor agonist CL316,243. Western blot analysis demonstrated that under basal conditions a distinct SELENOF protein band was detectable in WT cells, whereas it was completely absent in SELENOF-KO cells, confirming successful establishment of the KO model. Importantly, stimulation with CL316,243 for 6 h did not alter SELENOF protein expression in WT adipocytes, indicating that basal SELENOF expression is not regulated by β3-adrenergic receptor activation (Figure 8A).

To determine whether SELENOF deficiency affects early intracellular signaling responses to thermogenic stimulation, phosphorylation of the transcription factor CREB1 was examined following exposure to CL316,243 for 30 min. CL316,243 treatment markedly increased p-CREB1 levels in both WT and KO adipocytes. However, no significant differences were observed between genotypes, indicating that SELENOF deficiency does not impair proximal β3-adrenergic receptor signaling upstream of CREB1 activation (Figure 8B). To evaluate downstream thermogenic responses, the expressions of the thyroid hormone-activating enzyme DIO2 and the thermogenic effector protein UCP1 were examined following CL316,243 stimulation for 6 h. CL316,243 treatment significantly increased DIO2 and UCP1 protein expression in WT adipocytes, consistent with activation of the thermogenic program. In contrast, induction of both DIO2 and UCP1 was markedly attenuated in SELENOF-deficient adipocytes compared with WT adipocytes (Figure 8C). These findings indicate that although upstream β3-adrenergic signaling remains intact, SELENOF deficiency selectively attenuates execution of the downstream thermogenic transcriptional program.

Taken together with the in vivo observations of reduced FT_3_ availability and suppressed BAT DIO2 expression, these results support a model in which SELENOF deficiency attenuates local thyroid hormone-related signaling associated with UCP1 induction.

## 4. Discussion

### 4.1. SELENOF Deficiency Exacerbates HFD-Induced Metabolic Impairment in Female Mice

The present study demonstrates that SELENOF deficiency markedly increases susceptibility of female mice to HFD-induced metabolic disturbance. Compared with WT animals, SELENOF KO females exhibited more pronounced body weight gain, expansion of both ovWAT and iWAT depots, and enhanced BAT whitening following 16 weeks of HFD exposure, together with aggravated dyslipidemia, hyperleptinemia, and elevated serum ALT levels. In contrast, male mice subjected to identical dietary conditions did not display a comparable genotype-dependent amplification of metabolic alterations, indicating a sex-specific aspect of SELENOF action. No genotype-dependent differences were detected in glucose or insulin tolerance tests (GTT/ITT), suggesting that SELENOF deficiency did not measurably alter systemic glucose homeostasis in this model. The metabolic phenotype identified in SELENOF-deficient female mice is therefore characterized by adipose tissue expansion, adipokine imbalance, and disruption of central–peripheral energy communication, without concurrent systemic insulin resistance.

This temporal window is mechanistically informative. Instead of representing a secondary compensatory response to advanced metabolic decompensation, SELENOF deficiency appears to influence the stability of metabolic homeostasis during the transition from physiological adaptation to pathological remodeling under nutrient excess. Such regulatory roles are increasingly recognized as critical determinants of long-term metabolic trajectory and obesity progression [29,32]. Moreover, BAT whitening was exacerbated in SELENOF-deficient females, suggesting impaired adaptive thermogenesis during metabolic stress. Given that BAT dysfunction is thought to precede overt insulin resistance [42], these findings raise the possibility that SELENOF contributes to thermogenic resilience in females during the initial phase of metabolic challenge.

### 4.2. Adipose Tissue Remodeling and Hyperleptinemia Suggest That SELENOF Primarily Regulates Adipose Tissue Functional Quality Rather than Lipid Synthesis Capacity in Female Mice

Further analysis of peripheral adipose tissue revealed that SELENOF deficiency did not further enhance the HFD-induced upregulation of the lipogenic enzymes ACC and FAS. Instead, it significantly aggravated the suppression of the key lipolytic enzymes HSL and ATGL, accompanied by a marked increase in leptin mRNA and protein expression in ovWAT. These findings indicate that the adipose tissue expansion observed in SELENOF-deficient female mice is more likely attributable to impaired lipid mobilization and altered endocrine function of adipose tissue rather than to sustained activation of de novo lipogenesis. This distinction is mechanistically important. Rather than determining whether excess lipids are synthesized, SELENOF appears to contribute to the maintenance of adipose tissue adaptive flexibility under conditions of nutrient overload. In this context, SELENOF deficiency may compromise the ability of adipose tissue to preserve balanced lipolytic activity and appropriate adipokine secretion, thereby promoting an earlier transition from metabolically adaptive expansion toward dysfunctional adipose remodeling. Consistent with this interpretation, the further elevation of circulating leptin concentrations observed in SELENOF-deficient females occurred in parallel with systemic metabolic stress, suggesting that adipose-derived endocrine imbalance may contribute to whole-body metabolic dysregulation. Such alterations are characteristic of the transition from compensated adipose expansion to adipose tissue dysfunction, a process increasingly recognized as a critical determinant of metabolic disease progression.

Importantly, contemporary models of obesity-associated adipose pathology emphasize that metabolic deterioration is driven not only by increased fat mass per se but also by qualitative alterations in adipose tissue function, including impaired lipolysis, dysregulated adipokine secretion, and enhanced inter-organ metabolic signaling. Within this framework, the present findings support the interpretation that SELENOF participates in preserving adipose tissue endocrine and metabolic stability during early exposure to dietary excess, thereby limiting the propagation of systemic metabolic stress signals [46,47,48]. Taken together, these results suggest that SELENOF deficiency accelerates the transition from adaptive lipid storage toward dysfunctional adipose tissue remodeling, a shift that may represent one of the peripheral drivers of the central leptin resistance phenotype described in the subsequent section.

### 4.3. The Hypothalamic ER Stress–Leptin Resistance–Inflammation Axis as a Critical Signature of SELENOF Deficiency in Female Metabolic Impairment

One of the most mechanistically informative findings of the present study is that SELENOF deficiency markedly amplified HFD-induced hypothalamic ER stress while simultaneously aggravating impairments in leptin signaling and the activation of inflammatory pathways. Specifically, increased expression of GRP78, p-IRE1α, and p-PERK indicates enhanced activation of unfolded protein response signaling, whereas unchanged ObRb levels together with elevated SOCS3 expression and reduced STAT3 phosphorylation suggest that leptin signaling impairment occurred primarily at the post-receptor level.

Concurrently, the additional elevation of p-IKKα/β and p-p65 in SELENOF-deficient animals indicates activation of the NF-κB pathway within the hypothalamus. These signaling alterations are unlikely to be independent parallel effects. Rather, ER stress has been shown to activate the IKKβ/NF-κB pathway, which subsequently promotes SOCS3 expression and impairs STAT3 phosphorylation, thereby linking hypothalamic inflammation to leptin resistance [45]. A plausible explanation is that SELENOF normally supports ER protein quality control. SELENOF is retained in the ER through its association with UDP-glucose: glycoprotein glucosyltransferase (UGGT), and its thioredoxin-like redox-active domain has been proposed to reduce or isomerize disulfide bonds in UGGT-recognized misfolded glycoproteins [17,49,50]. Loss of this quality-control function could increase the burden of incompletely folded proteins and thereby lower the threshold for unfolded-protein-response activation during nutrient excess. However, this mechanism was not directly tested in hypothalamic cells in the present study and should therefore be regarded as a working hypothesis. This interpretation is consistent with emerging evidence demonstrating that ER proteostasis within hypothalamic metabolic neurons plays a critical role in maintaining leptin sensitivity. In particular, proper folding and trafficking of leptin receptor complexes in POMC neurons depend on intact ER quality-control mechanisms, whereas chronic ER stress promotes SOCS3-dependent suppression of STAT3 phosphorylation and contributes to the establishment of central leptin resistance [51,52]. Moreover, recent studies indicate that remodeling of the hypothalamic extracellular matrix and altered neuron–glia interactions further reinforce this stress-responsive signaling environment during obesity development [7,8,53,54,55].

Taken together, the present findings support the identification of SELENOF as a candidate ER-resident regulator of hypothalamic protein homeostasis and leptin responsiveness under HFD conditions. However, because the current conclusions are derived from systemic KO models, they do not yet establish a strictly causal sequence between enhanced ER stress and impaired leptin signaling. Future studies employing neuron-specific deletion strategies and functional rescue approaches will be required to determine whether SELENOF directly controls hypothalamic leptin receptor maturation or instead modulates upstream determinants of ER stress susceptibility.

The present findings further extend prior investigations into the metabolic implications of SELENOF deficiency. Li et al. [23] documented age-dependent glucose-metabolism abnormalities in young SELENOF-knockout mice, accompanied by disruption of redox homeostasis. By contrast, the present HFD model did not reveal genotype-dependent differences in GTT or ITT at the examined endpoint, but identified female-selective adipose, endocrine, hypothalamic, and BAT-associated alterations. Differences in age, dietary context, sex-specific analysis, and tissue endpoints may account for these distinct phenotypic profiles. Together with recent reviews of ER-resident selenoproteins in glucose and lipid metabolism [17], these observations indicate that the metabolic impact of SELENOF deficiency are context-dependent rather than restricted to a single metabolic pathway.

### 4.4. Impaired Thyroid Hormone Activation and Suppression of BAT Thermogenic Signaling as a Peripheral Mechanism of SELENOF Deficiency in Female Metabolic Regulation

At the peripheral level, the present study identified a coherent endocrine-thermogenic alteration associated with SELENOF deficiency. Although circulating concentrations of TSH and FT_4_ remained unchanged across experimental groups, both FT_3_ levels and the FT_3_/FT_4_ ratio were significantly reduced in SELENOF-deficient females exposed to HFD conditions. These findings suggest that SELENOF deficiency does not disrupt hypothalamic–pituitary–thyroid axis activity per se but instead selectively affects peripheral thyroid hormone activation. Consistent with this interpretation, the expression of the thyroid hormone-activating enzyme DIO2 in BAT was further reduced in SELENOF-deficient mice under HFD conditions, together with a corresponding decrease in UCP1 levels. Importantly, phosphorylation of CREB1, a key downstream component of β3-adrenergic receptor signaling, did not show an additional genotype-dependent reduction. This observation suggests that suppression of thermogenic signaling in SELENOF-deficient animals is unlikely to originate from defective upstream adrenergic signal transmission but may instead reflect attenuation of downstream thyroid hormone-related thermogenic execution.

Primary brown adipocyte experiments provided further support for this interpretation. While short-term stimulation with the β3-adrenergic receptor agonist CL316,243 induced comparable CREB1 phosphorylation in WT and SELENOF-deficient cells, prolonged stimulation resulted in markedly attenuated induction of DIO2 and UCP1 in KO adipocytes. Together, these findings indicate that SELENOF deficiency selectively interferes with activation of the DIO2-dependent local thyroid hormone signaling axis associated with thermogenic gene expression. This interpretation is consistent with the broader functional role of selenium-dependent proteins in endocrine metabolic regulation. Deiodinases represent critical determinants of tissue-specific T_3_ availability, and insufficient local T_3_ production in BAT has been shown to impair UCP1 induction and adaptive thermogenesis. In this context, the coordinated reduction in FT_3_ availability, DIO2 expression, and UCP1 levels observed in the present study supports a peripheral mechanism linking SELENOF deficiency with attenuated thermogenic responsiveness during early metabolic stress [17,23,24,56,57,58,59].

Collectively, these observations position SELENOF as a previously unrecognized contributor to tissue-specific thyroid hormone activation during metabolic stress. By limiting DIO2-dependent local T_3_ availability and downstream UCP1 induction, SELENOF deficiency may attenuate the thermogenic signaling program in BAT. Whether these molecular changes affect actual thermogenic capacity or whole-body energy expenditure warrants further investigation.

### 4.5. Sexual Dimorphism, Study Limitations, and Theoretical Implications of SELENOF-Dependent Metabolic Regulation

An additional important observation emerging from the present study is the pronounced sex-dependent nature of the metabolic phenotype associated with SELENOF deficiency. We observed no statistically significant difference in circulating estradiol concentrations between WT and KO female mice at the time of sampling. However, estradiol levels exhibit dynamic fluctuations across the estrous cycle, and female mice in this study were neither hormonally synchronized nor monitored for estrous stage. As a result, a single serum estradiol measurement is insufficient to exclude potential involvement of estrogen-dependent mechanisms. Moreover, no other sex hormones were assessed. Thus, the current dataset cannot distinguish whether the observed female-selective vulnerability arises from estrous-cycle-associated hormonal variation, estrogen-receptor signaling, local steroid metabolism, or differential responsiveness of thermogenic tissues.

Increasing evidence demonstrates that BAT exhibits marked sexual dimorphism at the levels of transcriptional regulation, endocrine sensitivity, and adaptive thermogenic activation [31,32,60,61,62]. Prior studies have demonstrated sex differences in BAT, including divergent hormonal responsiveness, transcriptomic profiles, and thermogenic adaptations between females and males [61,63,64]. The female-selective phenotype observed in the present study aligns with this established body of evidence but does not identify a single hormonal driver. Instead, it suggests that SELENOF-dependent metabolic regulation may functionally interact with sex-specific neuroendocrine and thermogenic programs.

Several limitations of the current study should nevertheless be considered when interpreting these findings. First, the use of a systemic SELENOF KO model does not permit precise discrimination between the relative contributions of central and peripheral SELENOF deficiency to the observed metabolic phenotype. Tissue-specific knockout and rescue approaches will be required to establish the causal hierarchy. Second, although the reduction in circulating FT_3_ levels and suppression of DIO2 expression in BAT strongly suggest impaired local thyroid hormone activation, direct functional measurements of adaptive thermogenesis—including indirect calorimetry, cold-exposure tests, core body temperature, and whole-body energy expenditure—were not performed. Thus, whether the molecular alterations translate into measurable impairment of thermogenic capacity remains to be determined. In addition, while primary brown adipocyte experiments support the existence of cell-autonomous thermogenic defects, further validation using tissue-specific rescue strategies will be required to determine whether SELENOF directly regulates brown adipocyte thermogenic competence in vivo. Third, the estrous cycle was not systematically monitored or synchronized in female mice, and other sex hormones were not measured. Although circulating estradiol levels did not differ significantly between genotypes under HFD conditions at the sampling time point, a single serum measurement cannot exclude contributions from estrous-cycle variation, local steroid metabolism, or hormone-receptor signaling.

Despite these constraints, the present findings support an integrative working model in which SELENOF may contribute to the coordination of central energy sensing and peripheral thermogenic signaling by maintaining hypothalamic ER proteostasis together with the DIO2-UCP1 axis in BAT. Disruption of this regulatory framework may increase susceptibility to metabolic imbalance, particularly in females. Further studies using larger cohorts, longitudinal designs, tissue-specific genetic models, and direct functional assays are required to establish the causal and translational relevance of SELENOF.

## 5. Conclusions

The present study provides preliminary evidence that SELENOF is associated with metabolic stability during HFD-induced metabolic stress and that its effects exhibit pronounced sex specificity. SELENOF deficiency increased the susceptibility of female mice to metabolic disturbances, including excessive adipose-tissue expansion, dyslipidemia, and hyperleptinemia, whereas comparable genotype-dependent effects were not observed in male animals.

Mechanistically, SELENOF deficiency was associated with coordinated alterations across central and peripheral metabolic regulatory compartments. At the hypothalamic level, loss of SELENOF aggravated endoplasmic reticulum stress signaling together with post-receptor leptin signaling impairment and activation of NF-κB-dependent inflammatory pathways, supporting disruption of central energy-sensing mechanisms. At the peripheral level, SELENOF deficiency selectively reduced circulating FT_3_ availability and suppressed the DIO2-UCP1 thermogenic axis in BAT, indicating compromised local thyroid hormone activation and adaptive thermogenic capacity. Complementary experiments in primary brown adipocytes further demonstrated that SELENOF loss attenuates downstream thermogenic program execution without impairing proximal β3-adrenergic signaling.

Collectively, these findings suggest a model in which SELENOF contributes to metabolic resilience by maintaining hypothalamic protein homeostasis, together with tissue-specific thyroid hormone activation in BAT, potentially linking central energy sensing with peripheral energy expenditure. This coordinated regulatory role expands the physiological framework of SELENOF action and provides a mechanistic basis for understanding sex-dependent susceptibility to metabolic dysfunction.

## Data Availability

The original contributions presented in this study are included in the article/Supplementary Materials. Further inquiries can be directed to the corresponding author.

## Supplementary Materials

The following supporting information can be downloaded at: https://www.mdpi.com/article/10.3390/biology15131017/s1. Supplementary Methods: Identification of SELENOF knockout mice. Figure S1: Identification of SELENOF knockout (KO) mice. (A) Genotypes of SE-LENOF KO and wild-type (WT) mice were determined by PCR and agarose gel electrophoresis. The SELENOF KO was obtained by deleting 29 bp in exon 2 using the CRISPR/Cas9 technique. In PCR analysis, three primers were used, with F1 and R flanking the deletion region and F2 located within the deleted segment. F1R amplified a product in both WT and KO mice, while F2R generated a band exclusively in WT mice. In KO mice, deletion of the 29-bp sequence abolished the F2 binding site, resulting in the loss of internal amplicon. (B) DNA sequencing of mouse genotypes. Sequencing of the targeted region in KO mice confirmed the precise 29-bp deletion in exon 2 of SELENOF, whereas the intact 29-bp sequence was retained in WT mice; Figure S2: White adipose accumulation and adipocyte hypertrophy in WT and SELENOF KO male mice fed a high-fat diet. (A) Representative images of epididymal white adipose tissue (eWAT) and inguinal white adipose tissue (iWAT) depots from male mice. (B) eWAT-to-body-weight and iWAT-to-body-weight ratios. (C) Representative hematoxylin and eosin (H&E) staining of eWAT and brown adipose tissue (BAT) from male mice. HFD feeding induced adipocyte enlargement in eWAT and lipid droplet accumulation in BAT, with no obvious differences between WT and KO mice. Scale bar, 100 μm. Data are presented as mean ± SD (*n* = 8–10 per group). Statistical differences were evaluated using one-way ANOVA followed by Tukey’s post hoc test. ** *p* < 0.01 vs. WT + ND group; ^##^ *p* < 0.01 vs. KO + ND group; Figure S3: Quantification of adipocyte size. (A) ovWAT from female mice. (B) BAT from female mice. (C) eWAT from male mice. (D) BAT from male mice. SELENOF deficiency exacerbated HFD-induced adipocyte enlargement in ovWAT and BAT of female mice, whereas no genotype-dependent differences were observed in male mice. For each group, a minimum of 100 adipocytes were measured. Data are presented as mean ± SD. Statistical differences were evaluated using one-way ANOVA followed by Tukey’s post hoc test. ** *p* < 0.01 vs. WT + ND; ^##^ *p* < 0.01 vs. KO + ND; ^ΔΔ^ *p* < 0.01 vs. WT + HFD; Figure S4: Effects of SELENOF deficiency on serum biochemical parameters in male mice following 16 weeks of HFD intervention. (A) Total cholesterol (TC). (B) Triglycerides (TG). (C) Free fatty acids (FFA). (D) Leptin. (E) Alanine aminotransferase (ALT). (F) Aspartate aminotransferase (AST). SELENOF deficiency had no significant effect on HFD-induced dyslipidemia, adipose endocrine activation, and hepatocellular stress in male mice. Data are presented as mean ± SD (*n* = 8–10). Statistical differences were evaluated using one-way ANOVA followed by Tukey’s post hoc test. * *p* < 0.05 and ** *p* < 0.01 vs. WT + ND group; ^##^ *p* < 0.01 vs. KO + ND group; Figure S5: Effects of SELENOF deficiency on circulating estradiol levels in female mice following 16 weeks of HFD intervention. Serum estradiol concentrations were not significantly altered by SELENOF deficiency under either normal diet or high-fat diet conditions. Data are presented as mean ± SD (*n* = 8–10). Statistical differences were evaluated using one-way ANOVA followed by Tukey’s post hoc test. Table S1: DNA Primer; File S1: The original Western blot images.

## Abbreviations

The following abbreviations are used in this manuscript:

ACC	acetyl-CoA carboxylase
ALT	alanine aminotransferase
AST	aspartate aminotransferase
ATGL	adipose triglyceride lipase
BAT	brown adipose tissue
CREB1	cAMP response element-binding protein 1
DIO2	type II iodothyronine deiodinase
ELISA	enzyme-linked immunosorbent assay
ER	endoplasmic reticulum
eWAT	epididymal white adipose tissue
E2	estradiol
FAS	fatty acid synthase
FFA	free fatty acids
FT_3_	free triiodothyronine
FT_4_	free thyroxine
FT_3_/FT_4_ ratio	ratio of free triiodothyronine to free thyroxine
GRP78	glucose-regulated protein 78
GTT	glucose tolerance test
HFD	high-fat diet
HSL	hormone-sensitive lipase
HSP90	heat shock protein 90
H&E	haematoxylin and eosin
IKK	IκB kinase
IRE1α	inositol-requiring enzyme 1 α
ITT	insulin tolerance test
iWAT	inguinal white adipose tissue
KO	knockout
ND	normal diet
NF-κB	nuclear factor kappa B
ObRb	long isoform of the leptin receptor
ovWAT	ovarian white adipose tissue
PERK	protein kinase R-like endoplasmic reticulum kinase
SOCS3	suppressor of cytokine signaling 3
STAT3	signal transducer and activator of transcription 3
TC	total cholesterol
TG	triglycerides
TSH	thyroid-stimulating hormone
UCP1	uncoupling protein 1
WAT	white adipose tissue
WT	wild type
