# Supplementary material for: Selenoprotein F Deficiency Drives Diet-Induced Metabolic Dysfunction in Female Mice by Aggravating Hypothalamic Endoplasmic Reticulum Stress
Source: Biology (Basel). 2026 Jun 26;15(13):1017. doi: 10.3390/biology15131017 (PMC13359464; doi:10.3390/biology15131017)
Supplement: Supplementary file 1 [file biology-15-01017-s001.zip › biology-4356789_Supplementary Materials File S1-The original Western blot images.pdf]

Supplementary Materials

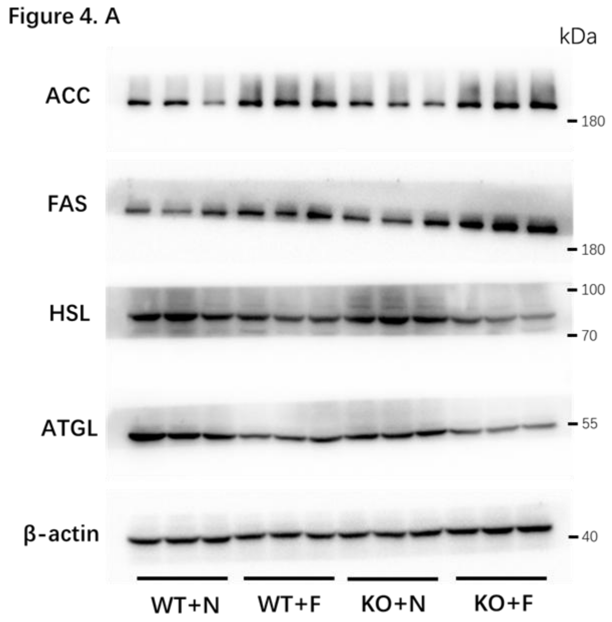

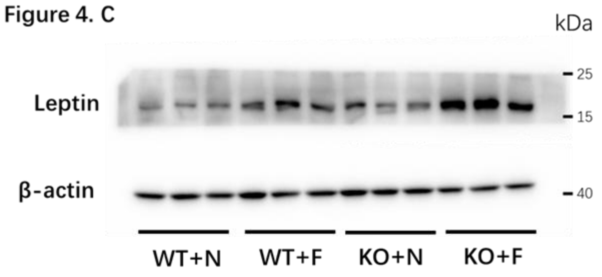

Figure 4A. Western blot analysis of lipogenesis and lipolysis markers in WAT.

Protein lysates from WAT were prepared using RIPA buffer supplemented with protease inhibitors (Solarbio, Beijing, China). Total protein concentrations were determined using a BCA protein assay kit (Elabscience). Equal amounts of protein were separated by SDS-PAGE and transferred onto PVDF membranes (Millipore, Burlington, MA, USA) using wet electroblotting. Membranes were blocked with blocking buffer (Boster, Wuhan, China) and incubated overnight at 4 °C with primary antibodies against the following proteins: ACC (Acetyl-CoA Carboxylase; 3662; 1:1000; Cell Signaling Technology), FAS (Fatty acid synthase; 3180; 1:1000; Cell Signaling Technology), HSL (Hormone-Sensitive Lipase; 4107; 1:1000; Cell Signaling Technology), ATGL (Adipose triglyceride lipase; A5126; 1:1000; ABclonal), and β-actin (AC026; 1:50000; ABclonal). Membranes were then incubated for 1 h at room temperature with HRP-conjugated secondary antibodies (Biosharp, Beijing, China; 1:5000 dilution). Protein signals were detected using an enhanced chemiluminescence substrate kit (Millipore) and visualized with a Tanon 5200 MultiImage System (Tanon, Shanghai, China). A prestained protein ladder (10–180 kDa; Thermo Scientific, catalog #26616) was used as a molecular weight marker.

Figure 4C. Western blot analysis of leptin protein expression in WAT.

Protein lysates were prepared and subjected to Western blot as described in Figure 4A. Membranes were incubated overnight at 4 °C with primary antibodies against leptin (DF8583, 1:500; Affinibody) and β-actin (AC026, 1:50000; ABclonal). Protein signals were detected as described above.

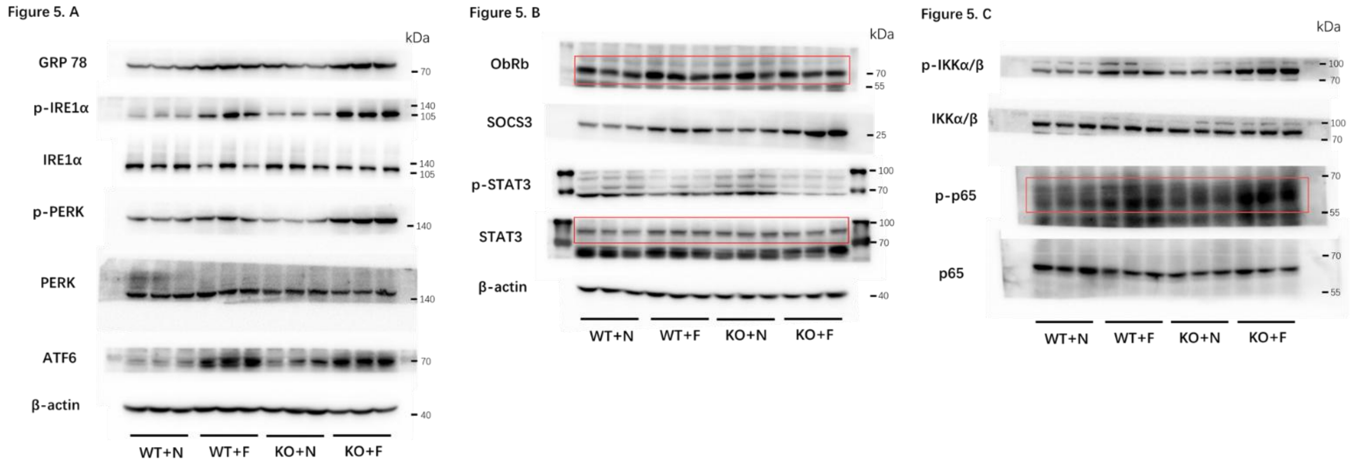

Figure 5A. Western blot analysis of ER stress markers in hypothalamus.

Protein lysates from hypothalamus were prepared using RIPA buffer supplemented with protease inhibitors. Total protein concentrations were determined using a BCA protein assay kit. Equal amounts of protein were separated by SDS-PAGE and transferred onto PVDF membranes using wet electroblotting. Membranes were blocked with blocking buffer (Boster, Wuhan, China) and incubated overnight at 4 °C with primary antibodies against the following proteins: GRP78 (ab108615, 1:1000; Abcam), p-IRE1α (Ser724, AF5842, 1:100; Beyotime), IRE1α (3294, 1:1000; Cell Signaling Technology), p-PERK (Thr982, AP1501, 1:1000; ABclonal), PERK (A27664, 1:1000; ABclonal), ATF6 (WL01153, 1:1000; Wanleibio), and β-actin (AC026, 1:50000; ABclonal). Membranes were then incubated for 1 h at room temperature with HRP-conjugated secondary antibodies (1:5000 dilution). Protein signals were detected using an enhanced chemiluminescence substrate kit and visualized with a Tanon 5200 MultiImage System. A prestained protein ladder (10–195 kDa; Servicebio, catalog #G2087) was used as a molecular weight marker.

Figure 5B. Western blot analysis of leptin resistance markers in hypothalamus.

Protein lysates were prepared and subjected to Western blot as described in Figure 5A. Membranes were incubated overnight at 4 °C with primary antibodies against ObRb (WL0162a, 1:1000; Wanleibio), SOCS3 (WL01364, 1:1000; Wanleibio), p-STAT3 (Ser727, WL06214, 1:1000; Wanleibio), STAT3 (WL01836, 1:1000; Wanleibio), and β-actin (AC026, 1:50000; ABclonal). Membranes were then incubated with HRP-conjugated secondary antibodies, and protein signals were detected as described in Figure 5A. A prestained protein ladder (10–180 kDa; Thermo Scientific, catalog #26616) was used as a molecular weight marker.

Figure 5C. Western blot analysis of inflammatory signaling markers in hypothalamus.

Protein lysates were prepared and subjected to Western blot as described in Figure 5A. Membranes were incubated overnight at 4 °C with primary antibodies against p-p65 (Ser536, 3033, 1:1000; Cell Signaling Technology), p65 (8242, 1:1000; Cell Signaling Technology), p-IKKα/β (Ser176/180, WLA0347, 1:1000; Wanleibio), IKKα/β (WL01900, 1:1000; Wanleibio), and HSP90 (WL01763, 1:1000; Wanleibio). Membranes were then incubated with HRP-conjugated secondary antibodies, and protein signals were detected as described in Figure 5A. A prestained protein ladder (10–180 kDa; Thermo Scientific, catalog #26616) was used as a molecular weight marker.

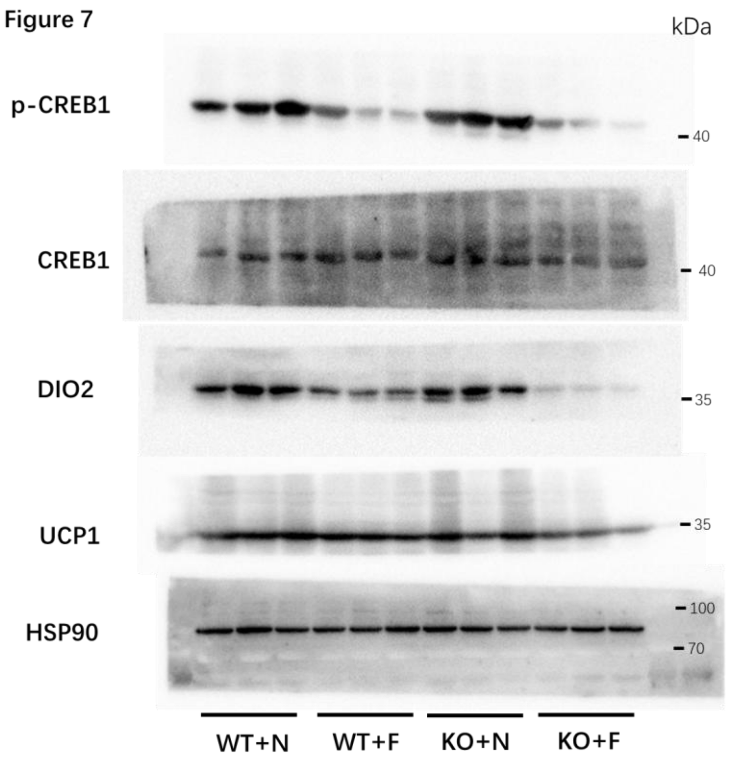

Figure 7. Western blot analysis of thermogenic markers in BAT.

Protein lysates from BAT were prepared using RIPA buffer supplemented with protease inhibitors. Total protein concentrations were determined using a BCA protein assay kit. Equal amounts of protein were separated by SDS-PAGE and transferred onto PVDF membranes using wet electroblotting. Membranes were blocked with blocking buffer and incubated overnight at 4 °C with primary antibodies against the following proteins: p-CREB1 (Ser133, WLA0644, 1:1000; Wanleibio), CREB1 (WL01848, 1:1000; Wanleibio), DIO2 (CSB-PA440740, 1:1000; Cusabio), UCP1 (A21979, 1:1000; ABclonal), and and HSP90 (WL01763, 1:1000; Wanleibio). Membranes were then incubated for 1 h at room temperature with HRP-conjugated secondary antibodies (1:5000 dilution). Protein signals were detected using an enhanced chemiluminescence substrate kit and visualized with a Tanon 5200 MultiImage System. A prestained protein ladder (10–180 kDa; Thermo Scientific, catalog #26616) was used as a molecular weight marker.

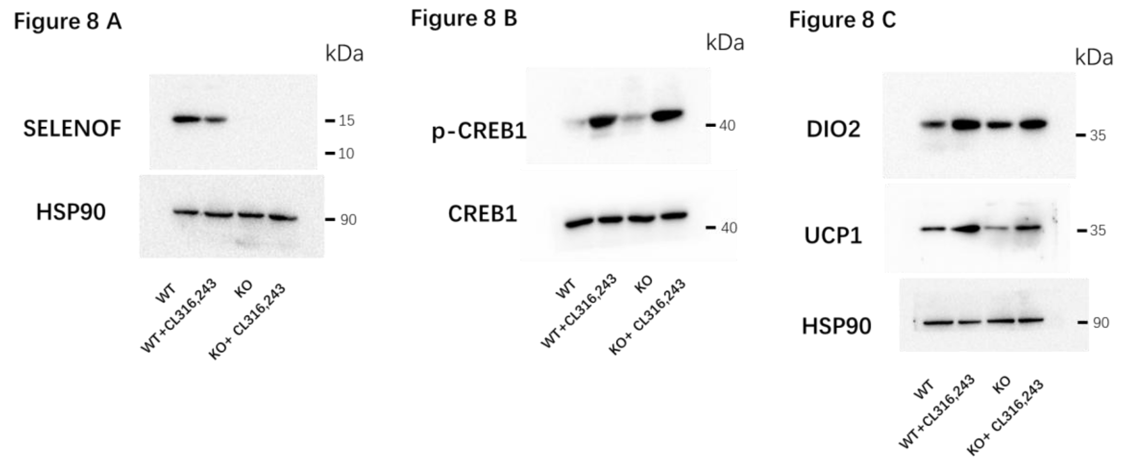

Figure 8A. Western blot analysis of SELENOF protein expression in brown adipocytes.

Protein lysates from brown adipocytes were prepared using RIPA buffer supplemented with protease inhibitors. Total protein concentrations were determined using a BCA protein assay kit. Equal amounts of protein were separated by SDS-PAGE and transferred onto PVDF membranes using wet electroblotting. Membranes were blocked with blocking buffer and incubated overnight at 4 °C with primary antibodies against SELENOF (1:500; Boster) and HSP90 (WL01763, 1:1000; Wanleibio). Membranes were then incubated for 1 h at room temperature with HRP-conjugated secondary antibodies (1:5000 dilution). Protein signals were detected using an enhanced chemiluminescence substrate kit and visualized with a Tanon 5200 MultiImage System. A prestained protein ladder (10–180 kDa; Thermo Scientific, catalog #26616) was used as a molecular weight marker.

Figure 8B. Western blot analysis of p-CREB1 and CREB1 in brown adipocytes.

Protein lysates were prepared and subjected to Western blot as described in Figure 8A. Membranes were incubated overnight at 4 °C with primary antibodies against p-CREB1 (Ser133, WLA0644, 1:1000; Wanleibio) and CREB1 (WL01848, 1:1000; Wanleibio). Membranes were then incubated with HRP-conjugated secondary antibodies, and protein signals were detected as described in Figure 8A.

Figure 8C. Western blot analysis of thermogenic markers DIO2 and UCP1 in brown adipocytes.

Protein lysates were prepared and subjected to Western blot as described in Figure 8A. Membranes were incubated overnight at 4 °C with primary antibodies against DIO2 (CSB-PA440740, 1:1000; Cusabio), UCP1 (A21979, 1:1000; ABclonal), and HSP90 (WL01763, 1:1000; Wanleibio). Membranes were then incubated with HRP-conjugated secondary antibodies, and protein signals were detected as described in Figure 8A.
