# Supplementary material for: Selenoprotein F Deficiency Drives Diet-Induced Metabolic Dysfunction in Female Mice by Aggravating Hypothalamic Endoplasmic Reticulum Stress
Source: Biology (Basel). 2026 Jun 26;15(13):1017. doi: 10.3390/biology15131017 (PMC13359464; doi:10.3390/biology15131017)
Supplement: Supplementary file 1 [file biology-15-01017-s001.zip › biology-4356789-Supplementary Materials.pdf]

Supplementary Information (SI)

Selenoprotein F deficiency drives diet-induced metabolic dysfunction in female mice by aggravating hypothalamic endoplasmic reticulum stress

This file includes:

A. Supplementary Methods

B. Supplementary Figures S1–S5

C. Supplementary Table S1

Supplementary Methods

Identification of SELENOF knockout mice. SELENOF knockout (KO) mice on a C57BL/6 background were maintained in our laboratory. Genotyping was performed at 4 weeks of age by PCR analysis of tail-tip DNA. Tail tips (approximately 2 mm) were collected, and genomic DNA was extracted using a commercial kit (D7283S; Beyotime, Shanghai, China). PCR was performed using the following primers: Forward Primer 1 (F1), 5′-AGGTCGTCTCCAGCACAG-3′; Forward Primer 2 (F2), 5′-CCTGACTTGCCCTTCCCT-3′; Reverse Primer (R), 5′-GGCAGAGTTTGCGTCAGA-3′. The cycling conditions were: initial denaturation at 95 °C for 5 min; 35 cycles of 95 °C for 30 s, 60 °C for 30 s, and 72 °C for 30 s; and a final extension at 72 °C for 5 min. PCR products (10 μL) were resolved by agarose gel electrophoresis, and genotypes were assigned based on amplicon size.

Supplementary Figure S1

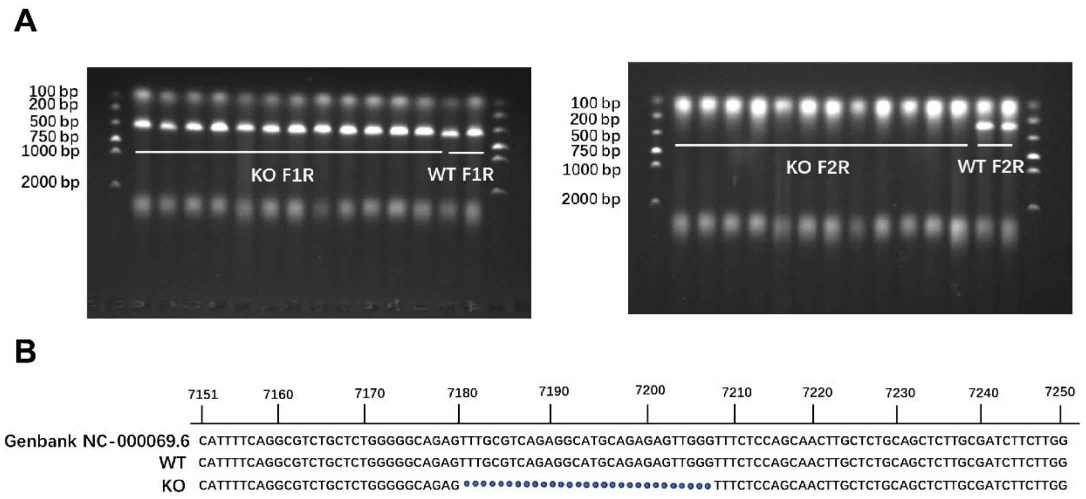

Figure S1. Identification of SELENOF knockout (KO) mice. (A) Genotypes of SELENOF KO and wild-type (WT) mice were determined by PCR and agarose gel electrophoresis. The SELENOF KO was obtained by deleting 29 bp in exon 2 using the CRISPR/Cas9 technique. In PCR analysis, three primers were used, with F1 and R flanking the deletion region and F2 located within the deleted segment. F1R amplified a product in both WT and KO mice, while F2R generated a band exclusively in WT mice. In KO mice, deletion of the 29-bp sequence abolished the F2 binding site, resulting in the loss of internal amplicon. (B) DNA sequencing of mouse genotypes. Sequencing of the targeted region in KO mice confirmed the precise 29-bp deletion in exon 2 of SELENOF, whereas the intact 29-bp sequence was retained in WT mice.

Supplementary Figure S2

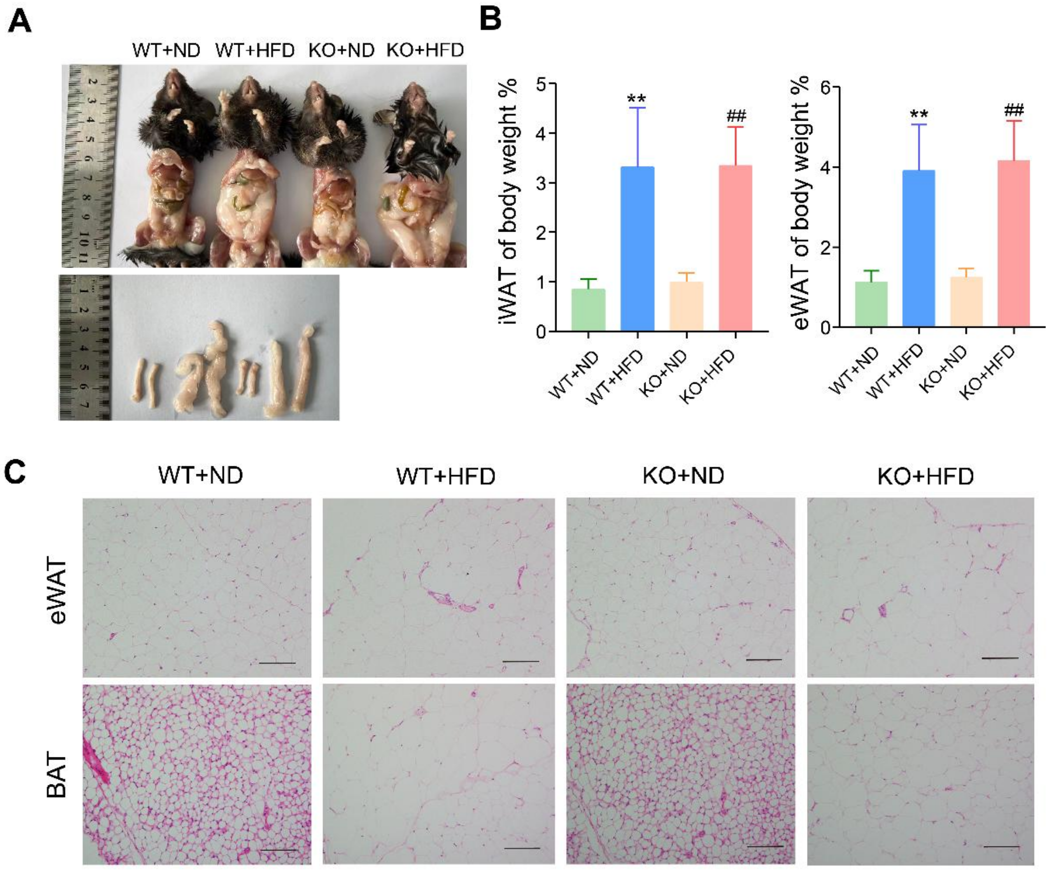

Figure S2. White adipose accumulation and adipocyte hypertrophy in WT and SELENOF KO male mice fed a high-fat diet. (A) Representative images of epididymal white adipose tissue (eWAT) and inguinal white adipose tissue (iWAT) depots from male mice. (B) eWAT-to-body-weight and iWAT-to-body-weight ratios. (C) Representative hematoxylin and eosin (H&E) staining of eWAT and brown adipose tissue (BAT) from male mice. HFD feeding induced adipocyte enlargement in eWAT and lipid droplet accumulation in BAT, with no obvious differences between WT and KO mice. Scale bar, 100 μm. Data are presented as mean ± SD (n = 8–10 per group). Statistical differences were evaluated using one-way ANOVA followed by Tukey's post hoc test. **p<0.01 vs. WT+ND group; $^{\#\#}p$<0.01 vs. KO+ND group.

Supplementary Figure S3

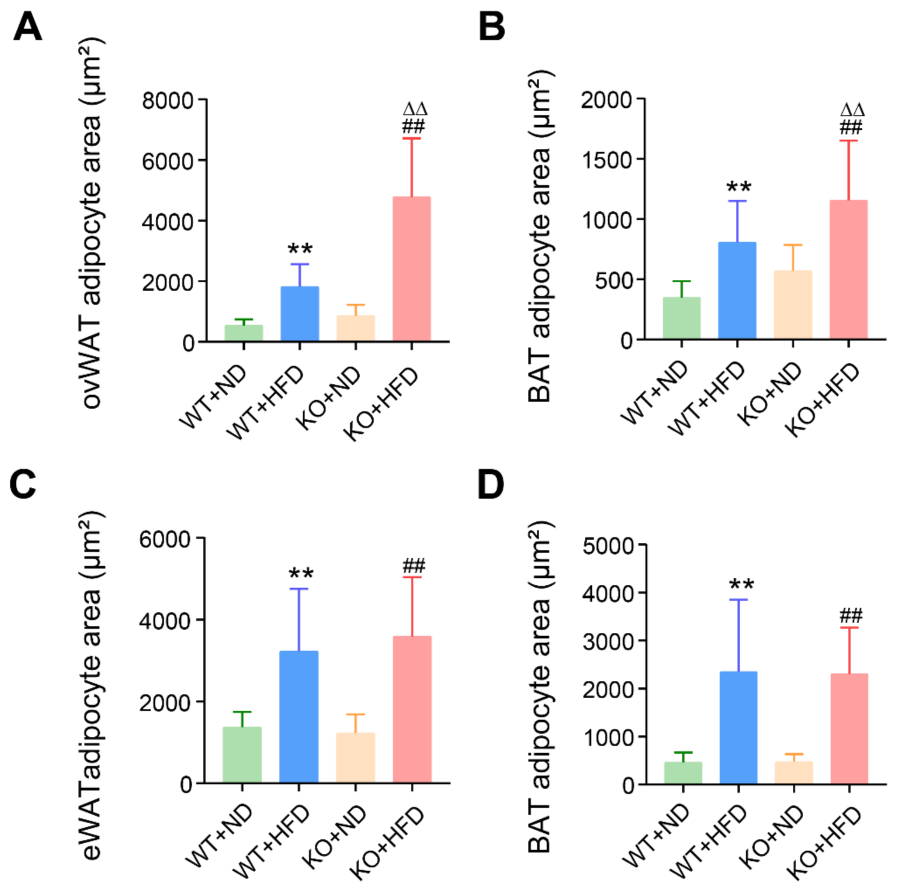

Figure S3. Quantification of adipocyte size. (A) ovWAT from female mice. (B) BAT from female mice. (C) eWAT from male mice. (D) BAT from male mice. SELENOF deficiency exacerbated HFD-induced adipocyte enlargement in ovWAT and BAT of female mice, whereas no genotype-dependent differences were observed in male mice. For each group, a minimum of 100 adipocytes were measured. Data are presented as mean ± SD. Statistical differences were evaluated using one-way ANOVA followed by Tukey's post hoc test. $^{**}p<0.01$ vs. WT+ND; $^{\#\#}p<0.01$ vs. KO+ND; $^{\Delta\Delta}p<0.01$ vs. WT+HFD.

Supplementary Figure S4

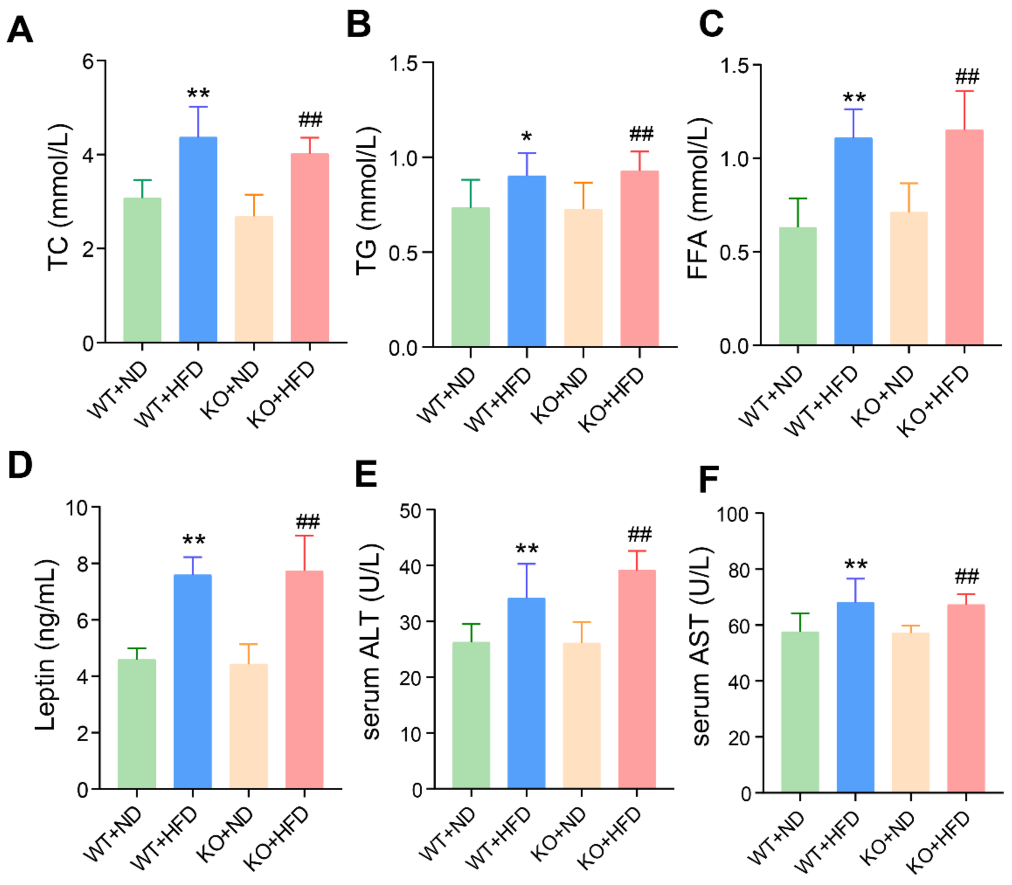

Figure S4. Effects of SELENOF deficiency on serum biochemical parameters in male mice following 16 weeks of HFD intervention. (A) Total cholesterol (TC). (B) Triglycerides (TG). (C) Free fatty acids (FFA). (D) Leptin. (E) Alanine aminotransferase (ALT). (F) Aspartate aminotransferase (AST). SELENOF deficiency had no significant effect on HFD-induced dyslipidemia, adipose endocrine activation, and hepatocellular stress in male mice. Data are presented as mean ± SD (n= 8–10). Statistical differences were evaluated using one-way ANOVA followed by Tukey's post hoc test. *$p<0.05$, **$p<0.01$ vs. WT+ND group; $^{\#\#}p<0.01$ vs. KO+ND group.

Supplementary Figure S5

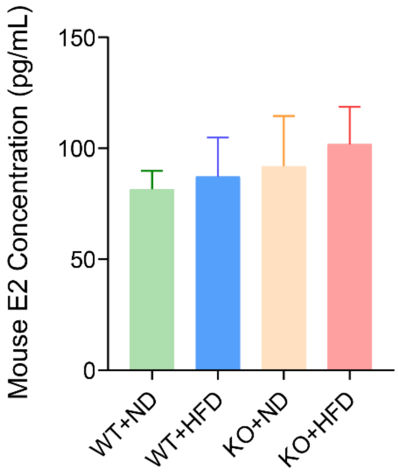

Figure S5. Effects of SELENOF deficiency on circulating estradiol levels in female mice following 16 weeks of HFD intervention. Serum estradiol concentrations were not significantly altered by SELENOF deficiency under either normal diet or high-fat diet conditions. Data are presented as mean ± SD (n= 8–10). Statistical differences were evaluated using one-way ANOVA followed by Tukey's post hoc test.

Supplementary Table S1

Table S1. DNA Primer

Gene	Primer (5'-3')
Leptin	Forward: ATTTCACACACGCAGTCGGT
	Reverse: ACATTTTGGGAAGGCAGGCT
β-actin	Forward: TTAGTTGCGTTACACCCTTTC
	Reverse: CTCGGCCACATTGTGAACT
